# Changes in Ultraviolet Radiation Exposure to the Ocular Region: A Population-Based Study

**DOI:** 10.3390/cancers11050719

**Published:** 2019-05-24

**Authors:** Ezekiel Weis, Sebastian Q. Vrouwe, David B. LeBaron, Matthew B. Parliament, Jerry Shields, Carol L. Shields

**Affiliations:** 1 Department of Ophthalmology, Faculty of Medicine and Dentistry, University of Alberta, Edmonton, AB T5H 3V9, Canada; svrouwe@hotmail.com (S.Q.V.); davidlebaron@gmail.com (D.B.L.); 2 Division of Ophthalmology, Department of Surgery, University of Calgary, Calgary, AB T2V 4R6, Canada; 3 Division of Radiation Oncology, Faculty of Medicine and Dentistry, University of Alberta, Edmonton, AB T6G 1Z2, Canada; matthew.parliament@albertahealthservices.ca; 4 Ocular Oncology Service, Wills Eye Hospital, Thomas Jefferson University, Philadelphia, PA 19107, USA; jerryshields@gmail.com (J.S.); carolshields@gmail.com (C.L.S.)

**Keywords:** ocular tumors, cutaneous melanoma, skin cancer, basal cell carcinoma, ultraviolet radiation, uveal melanoma, eye exposure

## Abstract

In contrast to the well-established association between ultraviolet radiation (UVR) exposure and skin cancers, the relationship between UVR and uveal malignant melanoma (UM) remains controversial. To address this controversy, we evaluated the incidence rates of cutaneous malignancies in the eyelids as a proxy for UVR exposure in the ocular region using a population-based cancer registry. Overall, 74,053 cases of eyelid basal cell carcinoma (BCC) and 7890 cases of melanoma over a 26-year period (1982–2007) were analyzed. The incidence of eyelid basal cell carcinoma and uveal melanoma remained stable, whereas other cutaneous areas demonstrated an increase in the rates. A comparability test demonstrated that BCC incidence trends were significantly different between the eyelid versus both chronically exposed (males *p* = 0.001; females *p* = 0.01) and intermittently exposed skin (males and females, *p* = 0.0002), as well as the skin of the face (males *p* = 0.002; females *p* = 0.02). Similarly, melanoma trends were significantly different between the UM group versus both chronically exposed cutaneous melanoma (CM) (males *p* = 0.001; females *p* = 0.04) and intermittently exposed CM (males *p* = 0.005), as well as facial skin CM (males and females *p* = 0.0002). The discrepancy of cancer incidence between tumors in the peri-ocular region versus the rest of the body suggests that the peri-ocular region might have a different or unique exposure pattern to ultraviolet radiation.

## 1. Introduction

Uveal (intra-ocular) malignant melanoma is the most common primary intra-ocular malignancy, and poses a significant risk of both vision loss and death, with up to 50% of patients dying of their disease [1,2]. It is also the second most common location for malignant melanoma to develop, after the skin [3,4]. Despite recent advances in treatment that often allow preservation of the eye (epi-scleral plaque brachytherapy, proton beam radiotherapy, stereotactic radiotherapy, local resection, and transpupillary thermotherapy) [5], there has been no improvement in survival outcomes [2,6]. This highlights the importance of understanding the underlying etiology of uveal malignant melanoma (UM) in order to best direct primary prevention.

There is a well-established causal link between ultraviolet radiation (UVR) exposure and both melanoma and non-melanoma skin cancers. [7,8,9,10] However, its role in the etiology of UM remains highly controversial [11,12,13,14,15,16,17,18,19,20,21]. In addition to sharing the same underlying malignant cell, the neural-crest-derived melanocyte, there are significant similarities in the high-risk phenotype for cutaneous malignancies and uveal melanoma, such as light-colored skin, the presence of nevi, and light-colored eyes [18,19,20,21,22,23,24]. Furthermore, incidence rates of uveal melanoma have been shown to decrease as skin pigmentation increases [25]. Other UV exposure factors such as latitude have demonstrated inconsistent results, with a meta-analysis of five articles demonstrating no significant association (OR = 1.08, 95% CI 0.67–1.74) [16]. Although the anterior segment (cornea, aqueous humor, and lens) absorbs the majority of UVR striking the eye, photochemical damage can still occur in the choroid [26]. Several studies assessing UM have not demonstrated UV signature mutations and significant differences in mutations between CM, conjunctival melanoma, and UM [27,28,29,30]. However, when controlling for intra-ocular location and its relation to UV exposure, UV signature genetic changes have been found in posterior uveal melanomas [31]. In addition, less common non-classical mutations found in uveal melanoma may be related to UV exposure [32]. The rising incidence of cutaneous melanoma (CM) [11,12,33,34] that accompanied changes in social/behavioral patterns resulting in intensifying sun exposure is in direct contrast to the incidence of UM that has been found to be stable [11,12,35,36,37] or decreasing over the same period [38,39]. This difference in incidence rates is one of the strongest criticisms of UVR as a causative factor in UM. Although these trends are indeed divergent, meaningful conclusions based on this observation must account for anatomical locations of lesions as well as their theoretically variable exposure patterns. Furthermore, characterizing the type of UVR exposure is important, as it has been shown that its pattern is critical in the development of CM whereby intermittent or recreational exposure, sunburns, childhood exposure, and chronic occupational exposure have variable associations [14,23,40,41].

This study was therefore designed to evaluate the changes in UVR exposure to the ocular region using BCC incidence as a proxy for UV exposure. Changes in incidence rates of basal cell carcinoma (BCC) of the eyelid—a common tumor with a well-described association with UVR—are used as a surrogate marker for changes in UVR exposure to the ocular region. These eyelid BCC rates are then formally contrasted to the rates of UM in an attempt to evaluate previously held notions on the association of UVR and UM. CM of the eyelid could not be used for this purpose, as its rarity would preclude robust statistical analysis.

## 2. Results

A total of 81,943 tumors (74,053 BCC and 7890 malignant melanoma) were identified over a 26-year period spanning 1 January 1982 to 31 December 2007. Of the BCC cases, 39,029 (52.7%) occurred in males, and 35,024 (47.3%) in females. Of the malignant melanoma (CM and UM) cases, 3891 (49.3%) occurred in males, and 3999 (50.7%) in females.

### 2.1. Age-Standardized Incidence Rates

Table 1 shows the average age-standardized incidence rates (ASIRs) of BCC and melanoma for four anatomical groupings (eyelid, chronically exposed skin, face, and intermittently exposed skin). For BCC, chronically UV exposed skin exhibited the largest average ASIR. Conversely, for melanoma, intermittently UV exposed skin displayed the largest average ASIR. Specifically, for peri-ocular tumors, the ASIR (per 100,000) for BCC of the eyelid was 6.72 ± 0.83 in males and 5.71 ± 0.70 in females, and for UM, 0.49 ± 0.21 in males and 0.46 ± 0.19 in females.

### 2.2. Incidence Trends

Table 2 shows trends in tumor incidence based on the average annual percent change (AAPC) derived from the underlying join point models of best fit. The incidence of BCC remained stable in the eyelid over the study period in both males and females. By contrast, significant upward trends in BCC incidence were observed in all other skin sites (chronically exposed skin, face, and intermittently exposed skin). The AAPC could not be calculated for CM of the eyelid due to small sample size.

A similar pattern of trends was observed for melanoma; the incidence of UM remained stable in both males and females, while significant positive trends were observed for CM in chronically and intermittently exposed skin. For CM in the facial skin, a statistically significant increase was seen in males, and a borderline increasing trend in incidence was seen in females (2.1%, 95% CI 0.0–4.3).

### 2.3. Comparability Test

**Figure 1:**
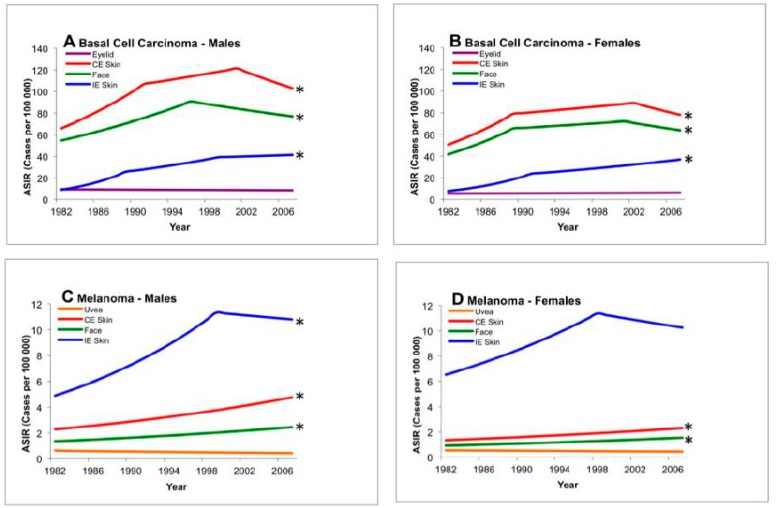
Test of parallelism comparing incidence trends of ocular and cutaneous tumors using registry data, 1982–2007. There is a statistically significant rising incidence in basal carcinoma and cutaneous melanoma of the face, intermittently exposed skin, and chronically exposed skin when compared to uveal melanoma or basal carcinoma of the eyelid. * Denotes non-parallel trend from baseline (eyelid or uvea, *p* < 0.05). ASIR: age-standardized incidence rate; CE: chronically exposed; IE: intermittently exposed.

Table 3 shows the results of the test of parallelism between anatomical regions, comparing peri-ocular/ocular (eyelid and uvea) and extra-ocular tumor incidence. BCC carcinoma change in incidence was significantly different than all other locations. Tests of parallelism comparing incidence trends for the eyelid versus other cutaneous regions revealed significantly different rates of change for chronically exposed skin (Figure 1: males *p* = 0.001; females *p* = 0.01), the face (Figure 1: males *p* = 0.002 and females *p* = 0.02), and intermittently exposed skin (Figure 1: males *p* = 0.0002; females *p* = 0.0002).

**Figure 2:**
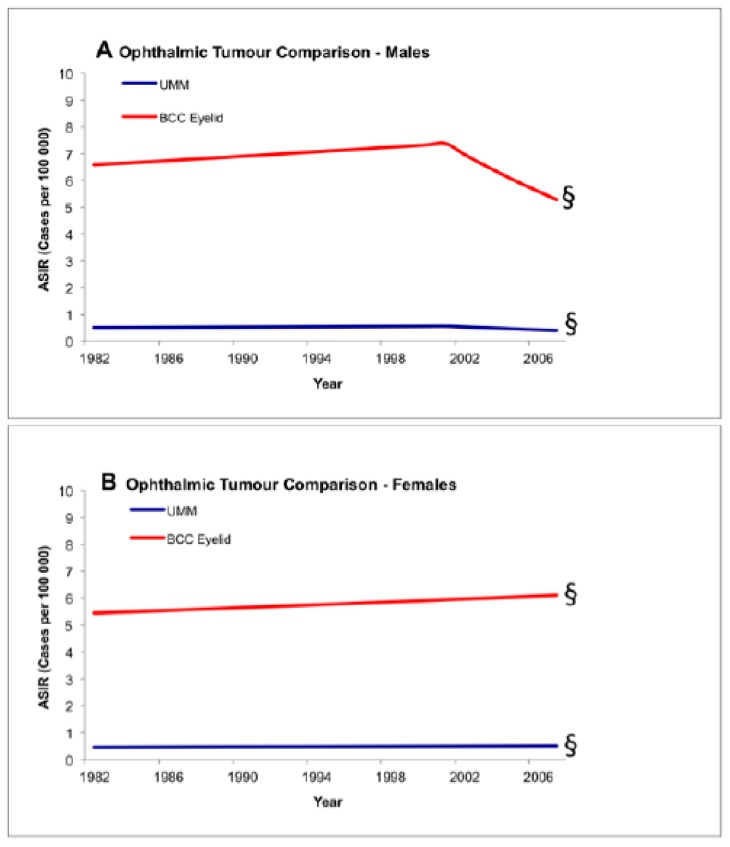
Test of parallelism comparing incidence trends between ocular tumors using registry data, 1982–2007. There is no statistically significant increase in the incidence of basal carcinoma of the eyelid and uveal melanoma. § Denotes parallel trend (*p* < 0.05). ASIR: age-standardized incidence rate; BCC: basal cell carcinoma; UMM: uveal malignant melanoma.

For melanoma, tests of parallelism comparing incidence trends for UM versus CM found significantly different rates of change. These changes were statistically significant for chronically exposed skin (Figure 2: males *p* = 0.001; females *p* = 0.04), the face (Figure 2: males *p* = 0.0002 and females *p* = 0.0002), and intermittently exposed skin (Figure 2: males *p* = 0.005). As mentioned previously, the rarity of CM of the eyelid precluded statistical analysis.

An additional test of parallelism was performed within the ocular groupings, comparing incidence trends between UM and BCC of the eyelid. Incidence changes between UM and BCC of the eyelid were not statistically different/occurred in parallel (Figure 2: males *p* = 0.08 and females *p* = 0.24).

## 3. Discussion

As sun exposure behaviors have changed, a strong association between the increasing incidence of cutaneous malignancies and UVR has been documented [11,12,33,34]. However, for UM, the absence of a similar increase in incidence over the same time period has been used as a principle argument against its association with UVR [11,12,35,36,37]. The validity of this argument is based on the premise that comparable increases in UVR exposure have occurred in the ocular region as in the rest of the body. Our study, utilizing the well-established association between BCC of the skin and UVR, developed a proxy variable that allowed for the assessment of changes in UVR exposure to the peri-ocular versus extra-ocular regions.

**Figure 3:**
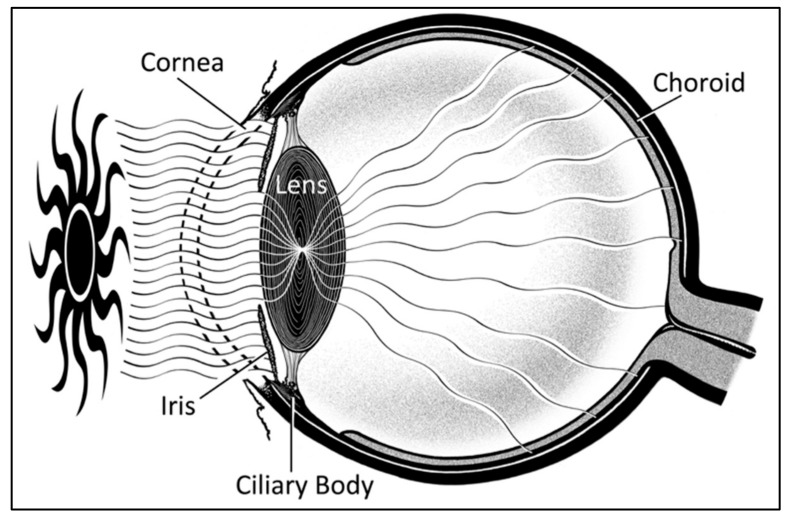
Sagittal schematic of the eye demonstrating ultraviolet exposure of iris and choroid, with sparing of the ciliary body due to protection by the iris.

UM is often considered as a single “intra-ocular” entity, yet it consists of three separate topographical locations including the choroid, ciliary body, and iris. All topographies have the neural-crest derived melanocyte within their cellular make-up, and thus carry its malignant potential. Choroidal melanoma comprises the majority (80%) of cases, followed by the ciliary body, and then the iris [11,12,42]. Due to its location behind the pigmented iris, the ciliary body likely receives significantly less UVR exposure [31,43], and therefore is less likely to be associated with UVR (Figure 3). Accordingly, its incidence would not be expected to vary with changing UVR exposure patterns. Unfortunately, ICD-O codes do not differentiate between the iris and ciliary body (C69.4). Thus, in an attempt to directly evaluate the role of UVR on exposed intra-ocular structures, only cases of choroidal melanoma were included in the analysis.

We report a stable trend in the incidence of eyelid BCC; however, there were upward trends for non-eyelid BCC (chronically and intermittently exposed skin, and the face). This divergence suggests that the ocular region displays a differential and unique exposure pattern to ultraviolet radiation that was not subject to the same increases seen in other body sites.

In a similar manner, incidence rates of UM were compared to rates of CM in non-ocular sites. Mirroring the eyelid BCC results, UM was found to have a stable incidence rate throughout the study period, while CM incidence increased in chronically and intermittently exposed skin. Incidence rates of eyelid BCC group were then directly compared to those of UM, revealing no statistical difference in the change of incidence rates over the study period.

Prior research is consistent with the findings of this study, although there has been no previous study quantitatively comparing peri-ocular malignancies to other anatomical locations [44,45,46,47,48]. A large population-based study in the United States analyzed incidence rates for BCC and squamous cell carcinoma during the years 1972–1973 and 1977–1978 and found that the incidence of both tumors increased in nearly all sites, with the exception of the eyelid, which appeared to be decreasing [44]. A registry-based study in the Netherlands (1975–1988) reported a fluctuating pattern of BCC incidence in the eyelids of females, while increases were observed for the face, neck, and scalp [45]. A large population-based study in Finland (1953–1997) reported an average increase of eyelid BCC over its entire period; however, incidence rates stabilized and began to decrease after 1983 [46]. Similarly, a smaller registry study from Singapore (1968–1995) also reported an average increase in all eyelid tumors (84% basal cell carcinoma) over the entire study period, but rates stabilized for males after 1978 and for females after 1983 [47]. Although not specific to BCC, a long-term registry-study in Sweden (1960–2004) found evidence of another UVR-implicated tumor—squamous cell carcinoma—increasing in all skin sites except the eyelid [48].

Although no formal statistical comparisons between UM and CM trends have been performed, several reports have suggested a difference [11,12], consistent with the findings of this study. A large population-based study from the United States (1974–1998) reported a stable annual percent change of UM incidence, while the incidence of CM increased significantly for both chronically exposed skin (face and ears, scalp and neck) and intermittently exposed skin (trunk and limbs) [11]. Similarly, a registry study from Denmark (1943–1997) reported a stable trend in the incidence of UM, with an increasing rate of CM in all skin sites, including the skin of the face, neck, and scalp [12].

Independent of cancer incidence studies, the notion that the ocular region displays a unique exposure pattern is furthered by a series of manikin, in vivo, and computational UVR studies [49,50,51,52,53,54]. Several investigators have employed manikins equipped with solar dosimeters localized across anatomical regions to quantify UVR doses, finding that the ocular region displayed relatively lower exposures compared to other parts of the face [49,50]. Using similar methods, others have demonstrated substantial variability of ocular UVR exposure depending on the use of hats and eyewear and environmental surface reflectivity [51,52]. Another study used computational methods to model UVR exposure at higher resolutions, revealing the importance of reflective properties intrinsic to the anatomy of the peri-ocular structures, calling into question prior dosimetry studies [53]. A review article summarized that the ocular region is different from the skin in other areas secondary to an increased component of reflected and scattered light, peak exposure varying due to the primary role of incident light, the protective effects of the geometry of the face, and back reflection from eye and sunglasses [54]. Although not affecting the eyelid, research has demonstrated that no UV light can pass through the lens after the age of 20 [26]. Taken together, these exposure studies suggest that ocular UVR exposure is unique and affected by many factors [55].

The strengths of this study included the use of high-quality registry data that covers a large population, and the novel approach of using eyelid BCC—the most common eyelid malignancy in Caucasian populations [56]—as a surrogate marker for UVR exposure to the ocular region. Although unlikely to bias our analyses, one limitation of our study was the exclusion of registry data classified topographically as “skin, not otherwise specified” and “overlapping lesion of the skin”; 242 cases of CM and 2343 cases of BCC were coded as such, and would have amounted to only 3.0% and 3.1% of the total number of cases, respectively. Another limitation is the absence of other relevant intra-ocular and ocular-adnexal tumors in our study, namely malignant melanoma of the eyelid, conjunctiva, and iris; these tumors were too rare to be subject to analysis, or the data were unavailable.

## 4. Materials and Methods

Research adhered to the tenets of the Declaration of Helsinki. Institutional Review Board approval was obtained.

### 4.1. The Alberta Cancer Registry

Alberta is a province in Western Canada with a population of 3.7 million [57]. It has a universal government-payer-based health care system and provincial cancer agency, providing care for all residents of the province. The Alberta Cancer Registry has been awarded gold certification from the North American Association of Central Cancer Registries for their 2006 incidence data based on completeness, timely reporting, and other measures of data quality [58].

### 4.2. Data Selection and Age Standardization

Cancer incidence data were collected over a 26-year period (1982–2007) for cutaneous BCC and cutaneous and uveal melanoma. Topographic areas were categorized to assess our hypothesis and acknowledge and allow comparisons with prior research differentiating incidence rates of various anatomical regions and their association with UV exposure. The following categories were retrieved (Table 4): uvea (choroid), eyelid, chronically exposed skin (lip, ear, scalp, neck, face including nose), unspecified parts of the face (not eyelid, lip, ear, scalp, neck), and intermittently exposed skin (trunk, limbs). Only malignant neoplasms stated or presumed to be primary (behavior code 3) were included (Table 3). UM is often considered as a single “intra-ocular” entity, yet it consists of three separate topographical locations, including the choroid, ciliary body, and iris. The age-standardized incidence rate (ASIR) was calculated separately for males and females, using the direct method with the United States year 2000 standard population [59]. Annual Alberta population figures by age and sex were obtained from the provincial government ministry of health. Non-Alberta residents were excluded from the dataset. Ethics approval was obtained from the Alberta Cancer Research Ethics Committee, and the approval file was received.

### 4.3. Joinpoint and the Comparability Test for Differences between Groups

Statistical analysis was performed with the Joinpoint Regression Program (National Cancer Institute, Version 3.4.3) [60,61]. Briefly, for each tumor grouping’s log-linear model, an average annual percent change (AAPC) in incidence was calculated for the entire study period [62]. AAPCs are a weighted average of the final model’s individual trend segments, with a 95% confidence interval based on a normal distribution that shows if the average trend is significant (increasing or decreasing) or non-significant from zero (stable). A comparability test known as the test of parallelism [63] was performed in Joinpoint to contrast incidence trend data between different anatomical groupings. The significance level was set at *p* < 0.05.

## 5. Conclusions

The controversial association between UVR and UM has not yet been resolved. A primary argument against this association is the observation that while CM time trends in incidence have risen with increasing UV exposure in the past decades, UM rates have remained stable. This argument assumes that changes in UVR exposure patterns of the ocular region are similar to those of the cutaneous regions that have driven the increasing incidence rates. The results of our study refute this premise by demonstrating that the ocular region has not been subject to an increase in UVR exposure with changing sun behavior patterns.

## Figures and Tables

**Table 1:** Average age-standardized incidence rates (ASIRs) for ophthalmic and non-ophthalmic basal cell carcinoma and melanoma, 1982–2007.

Anatomical Grouping	Males		Females		Combined	
	*n*	Average ASIR ± SE	*n*	Average ASIR ± SE	*n*	Average ASIR ± SE
**Basal cell carcinoma**						
Eyelid	1867	6.72 ± 0.83	1844	5.71 ± 0.70	3711	6.13 ± 0.53
Chronically exposed skin	27,934	102.21 ± 3.29	24,819	77.52 ± 2.58	52,753	88.41 ± 2.03
Face	20,803	76.11 ± 2.84	20,296	63.56 ± 2.34	41,099	69.05 ± 1.80
Intermittently exposed skin	9228	29.91 ± 1.65	8361	24.15 ± 1.37	17,589	26.69 ± 1.05
**Melanoma**						
Uvea	137	0.49 ± 0.21	144	0.46 ± 0.19	288	0.47 ± 0.14
Eyelid	12	0.04 ± 0.04	18	0.05 ± 0.04	30	0.04 ± 0.04
Chronically exposed skin	905	3.33 ± 0.60	573	1.71 ± 0.37	1478	2.42 ± 0.33
Face	464	1.81 ± 0.45	372	1.13 ± 0.30	836	1.42 ± 0.26
Intermittently exposed skin	2849	8.68 ± 0.86	3282	9.36 ± 0.86	6131	8.99 ± 0.61

**Table 2:** Incidence trends and the average annual percent change (AAPC) for ophthalmic and non-ophthalmic basal cell carcinoma and melanoma, 1982–2007.

Anatomical Grouping	Males		Females	
	Trend	AAPC (95% CI)	Trend	AAPC (95% CI)
**Basal cell carcinoma**				
Eyelid	Stable	−0.4 (−1.1 to 0.4)	Stable	0.6 (−0.1 to 1.2)
Chronically exposed skin	Increasing	1.8 * (0.9 to 2.8)	Increasing	1.7 * (1.1 to 2.4)
Face	Increasing	1.4 * (0.7 to 2.1)	Increasing	1.7 * (0.9 to 2.5)
Intermittently exposed skin	Increasing	6.3 * (5.1 to 7.6)	Increasing	6.4 * (5.2 to 7.7)
**Melanoma**				
Uvea	Stable	−1.4 (−3.6 to 0.8)	Stable	−0.9 (−2.9 to 1.2)
Eyelid	§		§	
Chronically exposed skin	Increasing	3.0 * (1.9 to 4.1)	Increasing	2.2 * (0.5 to 3.9)
Face	Increasing	2.4 * (1.0 to 3.8)	Stable	2.1 (−0.0 to 4.3)
Intermittently exposed skin	Increasing	3.2 * (1.9 to 4.5)	Increasing	2.0 * (0.6 to 3.3)

§ Unable to calculate due to low numbers; * Statistically significant difference.

**Table 3:** Test of parallelism between anatomic groupings for basal cell carcinoma and melanoma, 1982–2007.

Test of Parallelism		Result	
Group 1	Group 2	Males	Females
**Basal cell carcinoma**			
Eyelid vs.	Chronically exposed skin	* Not parallel (*p* = 0.001)	* Not parallel (*p* = 0.01)
	Face	* Not parallel (*p* = 0.002)	* Not parallel (*p* = 0.02)
	Intermittently exposed skin	* Not parallel (*p* = 0.0002)	* Not parallel (*p* = 0.0002)
Chronically exposed skin vs.	Intermittently exposed skin	* Not parallel (*p* = 0.0002)	* Not parallel (*p* = 0.0002)
**Melanoma**			
Uvea vs.	Chronically exposed skin	* Not parallel (*p* = 0.001)	* Not parallel (*p* = 0.04)
	Face	* Not parallel (*p* = 0.002)	* Not parallel (*p* = 0.03)
	Intermittently exposed skin	* Not parallel (*p* = 0.005)	Parallel (*p* = 0.06)
Chronically exposed skin vs.	Intermittently exposed skin	Parallel (*p* = 0.81)	Parallel (*p* = 0.46)
**Inter-ophthalmic**			
Uveal malignant melanoma vs.	Eyelid basal cell carcinoma	Parallel (*p* = 0.08)	Parallel (*p* = 0.24)

* Statistically significant change over time.

**Table 4:** Topographical and morphological tumor groups for data retrieval, using the International Classification of Diseases for Oncology, 3rd Edition (ICD-O-3) histologic criteria.

Name	ICD-O Code	ICD-O Category	ICD-O Description
**Topographical group criteria**			
Uvea	C69.3	Eye and adnexa	Choroid
Eyelid	C44.1	Skin	Eyelid
Chronically exposed skin	C44.0, C44.2–C44.4	Skin	Skin of lip NOS, external ear, skin of other and unspecified parts of the face, skin of scalp and neck
Face	C44.3	Skin	Skin of other and unspecified parts of the face
Intermittently exposed skin	C44.5–C44.7	Skin	Skin of trunk, skin of upper limb and shoulder, skin of lower limb and hip
**Morphological tumor criteria**			
Basal cell carcinoma	8090/3-8094/3, 8097/3, 8098/3	Basal cell neoplasms	Basal cell carcinoma, NOS; multifocal superficial basal cell carcinoma; infiltrating basal cell carcinoma, NOS; basal cell carcinoma, fibroepithelial; basosquamous carcinoma; basal cell carcinoma, nodular; adenoid basal cell carcinoma
Malignant melanoma	8720/3–8723/3, 8730/3, 8740/3–8746/3, 8761/3, 8770/3–8774/3,8780/3	Nevi and melanomas	Malignant melanoma, NOS; nodular melanoma; balloon cell melanoma; malignant melanoma, regressing; amelanotic melanoma; malignant melanoma in junctional nevus; malignant melanoma in precancerous melanosis; lentigo maligna melanoma; superficial spreading melanoma; acral lentiginous melanoma, malignant; desmoplastic melanoma, malignant; mucosal lentiginous melanoma; malignant melanoma in giant pigmented nevus; mixed epithelioid and spindle cell melanoma; epithelioid cell melanoma; spindle cell melanoma, NOS; spindle cell melanoma, type A; spindle cell melanoma, type B; blue nevus, malignant

Only choroidal melanomas were included in this study.
